# Sports fanaticism as a disease: a Corpus-based study of metaphors in Saudi newspapers

**DOI:** 10.3389/fpsyg.2023.1286395

**Published:** 2024-01-04

**Authors:** Yahya Abdu A. Mobarki, Fahad Alzahrani

**Affiliations:** ^1^English Department, Faculty of Arts and Humanities, Jazan University, Jizan, Saudi Arabia; ^2^Languages and Translation Department, College of Education and Arts, University of Tabuk, Tabuk, Saudi Arabia

**Keywords:** systematic metaphor, fanaticism, disease, Saudi Arabia, sports, newspapers

## Abstract

Sports fanaticism seems to be a social and national concern in Saudi Arabia. This paper aims: 1) to identify the metaphorical manifestations and highlight the discursive construction of disease as associated with sports fanaticism in a corpus of Saudi newspapers articles; and 2) to seek plausible explanations for the emergence and use of disease metaphors in newspapers articles addressing sports fanaticism. King Abdulaziz City for Science and Technology Arabic Corpus (KACSTAC) served the data for the current study. The analytical procedures were informed by the discourse dynamics approach and metaphor-led discourse analysis. Findings show that sports fanaticism could be associated with the following systematic metaphors: 1) disease, in general; 2) disease causes; 3) disease symptoms; 4) disease evaluations; and 5) disease needs for medical solutions, interventions, treatments, and/or prescriptions. The discussion evokes a number of aspects: sports fanaticism emerges as a key topic; disease metaphors seem to be shaped and developed by the societal context and the nature of newspapers texts and discourse; they are enriched and influenced by the discourse situation and the needs to jointly construct and communicate intense experiences through specific evaluations and referential functions, which have a powerful resonance for the Saudi national and social levels; the use of disease metaphorical frames can structure our understanding and can simplify the concept of fanaticism.

## 1 Introduction

The beauty of language use lies in the ability to capture meaning within any context. Meanings might be expressed literally with the use of language to describe them, and they could be expressed figuratively by using imagery and context descriptions (Stern, 2000). One way to express meaning is by integrating conceptual imagery with language; consequently, this could generally be defined as metaphor use in linguistic terms. According to Lakoff and Johnson (1980) and Kövecses (2010), metaphor is defined as comprehending one experiential or conceptual domain (i.e., target domain) in terms of another experiential or conceptual domain (i.e., source domain). Metaphors are used in many languages. They aid in facilitating communication and play a role in shaping people’s way of thinking (Thibodeau et al., 2017). People use metaphors as a means to explain shared meanings and they could be culturally specific (Kövecses, 2005). According to Cameron et al. (2009), metaphors show the relationships between language and conceptual thought. Thus, metaphor is an integral component of human communication (MacArthur and Oncins-Martínez, 2012).

Linguistic metaphors are used in ways that may portray positive or negative connotations. One of the ways in which metaphors are utilized for negative descriptions and concepts related to intolerance are within the domain of sports and sport fanatics. Additionally, sports fanaticism and its metaphors have escaped systematic analysis in languages and cultures across the globe, in general, and Arabic, in particular. Therefore, the current study presents an unprecedented analysis of disease metaphors linked with sports fanaticism as a discursively constructed social and national problem.

This study has the following objectives:

- To highlight the discursive and metaphorical construction of sports fanaticism as disease in the Saudi newspapers.
- To identify the metaphorical frames through which sports fanaticism is associated with disease.
- To discuss the implications and applications of the aforementioned discursive construction and metaphorical frames.

The following research questions were raised to guide the study:

1. What are the metaphorical manifestations of disease associated with sports fanaticism?
2. What are the plausible explanations for the emergence and use of disease metaphors in newspaper articles addressing sports fanaticism?

The significance of this research resides in the following: First, research on metaphor, as Magaña and Matlock (2018) mentioned, focused overwhelmingly on English. With the body of available research, little is yet known about metaphor in Arabic contexts utilizing most recently developed approaches and methodologies. Also, it appears that there is a scarcity of research on topics related to sports fanaticism, metaphor, and the sociocultural and critical associations reflected on linguistic and/or conceptual levels of discourse. As such, the current research is expected to uncover new research directions for the scientific scholarship on metaphor. Second, this research celebrates the empricity embraced in the scholarship and research on metaphor. That is, this study carries the significance of studying metaphor in its actual usages and contexts using real-world data obtained from public discourse and corpora. Thus, this study is expected to provide “an empirical contribution to the study of metaphor,” which “contributes to our understanding of the social realities constructed in the areas of life” (Zinken and Musolff, 2009, p. 1), including sports and discourses of sports. The following section presents the theoretical framework and previous research.

## 2 Theoretical framework and previous research

### 2.1 Discourse dynamics approach and metaphor-led discourse analysis

Previous research on metaphors has been conducted through the lens of several theoretical approaches and conceptual frameworks (see Semino and Demjén, 2017 for a recent comprehensive review). The present study draws mainly on the discourse dynamics approach and metaphor-led discourse analysis as proposed and developed by Cameron (2003, 2004, 2007, 2008), Cameron et al. (2009). Cameron et al. (2009, p.68) resist the ideas that linguistic metaphor and conceptual metaphor are “top-down instantiation[s] from thought to language” as primarily assumed in Conceptual Metaphor Theory (CMT; Lakoff and Johnson, 1980; see Kövecses, 2010). Rather, they suggest that the connection is “one of interaction between language and thinking” and that “what is said both reflects and affects thinking” (Kövecses, 2010, p. 68). Therefore, this approach highlights the central significance of context and the dynamic nature and interaction between language, thought, and context, and their mutual influence on the emergence, development, and use of figurative language and imagery in discourse and communication.

A number of concepts are central to this approach: *key topic* (or *tenor*), *vehicle* (or *vehicle terms*), *linguistic metaphor*, *vehicle grouping*, and *systematic metaphor*. The key topic “is the real world referent of the vehicle word or phrase” (Cameron et al., 2009, p. 74). Vehicle terms are the words and phrases that carry incongruent semantic content or anomalous meaning compared to their more basic meaning. A linguistic metaphor refers to the actual and contextualized use of metaphors in language. Linguistic metaphors neither refers to metaphors in thought nor to linguistic instantiations of conceptual metaphors. In the discourse dynamics approach, the analyst develops vehicle groupings for the vehicle terms; each vehicle grouping captures the overarching and “essential semantic meaning” (Cameron et al., 2009, p. 75) for a group of vehicle terms. The result is then called a systematic metaphor. According to Cameron et al. (2009, p. 78), “the systematic metaphor is the dynamic collection of connected linguistic metaphors, a trajectory from one metaphor to the next over the dynamics of [discourse].” It is not a conceptual metaphor; at least, it is different theoretically and ontologically.” Section 3 (data and methods) and section 4 (findings and analysis) provide further empirical illustrations and examples for the previously described concepts.

According to Kövecses (2010), see Lakoff and Johnson (1980), two unique names representing two special domains are usually reported in the literature on metaphors research. The source domain is the domain from which we derive metaphorical terms to comprehend another domain. The target domain is the domain we attempt to comprehend by utilizing the source domain. Source domains are more tangible and concrete (e.g., WAR and JOURNEY), while target domains are more abstract and complex (e.g., LIFE and LOVE). Since this study investigates the disease metaphor of sports fanaticism, section 2.2 discusses disease and disease metaphors in the literature.

### 2.2 Disease and disease metaphors in the literature

The goal of this section is to present the current literature on disease metaphors. The reasons are as follows: The literature shows that disease metaphors have been widely researched and a number of themes seem to evolve with systematic significance.

To begin with, disease and disease metaphors have received scholarly treatments in various different languages: Of course, English is setting on the top most researched languages related to such phenomena (see for instance, Sontag, 1979, 1989; Semino et al., 2004, 2015, 2018; Potts and Semino, 2019). Other investigated languages include: Spanish (Landtsheer, 2009; Negro, 2016; Magaña and Matlock, 2018; Oster, 2019; Sabucedo et al., 2020), German (Oster, 2019), Ukrainian (Dilai and Serafin, 2019), French (Perrez and Reuchamps, 2014; Negro, 2016), Dutch (Perrez and Reuchamps, 2014), Italian (Wehling, 2016), Persian (Bakhtiar, 2017), Russian (Pinelli, 2016), Greek (Tsakona, 2012), Brazilian Portuguese (Pelosi et al., 2014; Ribeiro et al., 2018), Arabic ( Zibin, 2020; Abaalalaa and Ibrahim, 2022; Zibin and Hamdan, 2023), Chinese (Chiang and Duann, 2007), and several languages as in Olza et al. (2021), Brugman et al. (2022), and Pérez-Sobrino et al. (2022). Thus, one can say that disease metaphors have been investigated in several different languages.

Disease and disease metaphors have been studied using different research designs and utilizing various theoretical and conceptual orientations, frameworks, and methodologies. Scholars used experimental, or more descriptive and interpretative, research designs. They also utilized real world discourse and linguistic data either from available and accessible corpora and/or constructed (or collected) data (or corpora). Analysts’ introspections have been valuable in the discussions of very few scholars (e.g., Kövecses, 2000). On one hand, for instance, Thibodeau and Boroditsky (2011), Hendricks et al. (2018), Lu and Schuldt (2018), and Scherer et al. (2015) conducted experimental studies through which aspects of disease and relevant influencing factors were highlighted and discussed. On the other hand, descriptive and interpretative studies guided by the discourse analytic and/or cognitively-oriented scientific traditions pervade investigations of disease and disease metaphors. Potts and Semino’s (2019) study of cancer as a metaphor in contemporary English presented a nice illustration for the use of a combination of notions and tools from discourse analysis, corpus linguistics, and Conceptual Metaphor Theory (CMT).

First, to use the terminology of CMT (Lakoff and Johnson, 1980; see Kövecses, 2010), the literature shows that disease has been researched, analyzed, and discussed either as a source domain and/or as a target domain (Potts and Semino, 2019). With respect to disease/illness as a target domain (i.e., disease/illness/types-names of specific diseases/illnesses is something), the literature documents different metaphors for several illnesses, to name just a few: COVID-19 pandemic (Bates, 2020; Craig, 2020; Sabucedo et al., 2020; Olza et al., 2021; Brugman et al., 2022; Pérez-Sobrino et al., 2022), Avian flu (Hanne and Hawken, 2007; Nerlich and Halliday, 2007; Koteyko et al., 2008), Zika virus (Lu and Schuldt, 2018; Ribeiro et al., 2018), severe acute respiratory syndrome (SARS; Washer, 2004; Larson et al., 2005; Wallis and Nerlich, 2005; Chiang and Duann, 2007; Hudson, 2008), depression (Hendricks et al., 2018), diabetes and heart disease (Hanne and Hawken, 2007), MRSA-Superbug (Nerlich and Koteyko, 2009), foot and mouth disease (Nerlich, 2004; Larson et al., 2005), ebola (Balteiro, 2017), and HIV/AIDs (Sontag, 1989; Hanne and Hawken, 2007). Metaphors of general concepts such as Disease, illness, or medicine have received linguistic attention (Gwyn, 1999; Mongoven, 2006; Goatly, 2007; Periyakoil, 2008).

A substantial literature shows that metaphors for cancer are the most frequently investigated among all kinds of illnesses (Sontag, 1979; Bowker, 1996; Semino et al., 2004, 2015, 2018; Hanne and Hawken, 2007; Williams Camus, 2009; Hauser and Schwarz, 2015, 2019; Flusberg et al., 2018; Hendricks et al., 2018; Magaña and Matlock, 2018; Wackers et al., 2021; Abaalalaa and Ibrahim, 2022). Military and war metaphors (or violence and combative metaphors) are the most commonly reported source domains for diseases or illnesses as target domains. The following example “cancer was the *enemy to beat*” underlines the metaphor: CANCER/DISEASE IS AN ENEMY/WAR. There is also an increasing prevalence of journey metaphors (e.g., “…accompanying me on this *journey”*’ CANCER/DISEASE IS A JOURNEY), (natural) disasters (e.g., “SARS *burns out*…” SARS IS A FIRE), sports metaphors (e.g., “overcoming cancer is very similar to *running a race*” CANCER/DISEASE IS A RACE), and machine metaphors (e.g., “cancer cells *drive* the disease” CANCER IS A MACHINE).

With respect to disease/illness as a source domain (i.e., SOMETHING IS DISEASE/ILLNESS/TYPES-NAMES OF SPECIFIC DISEASES/ILLNESSES), it is substantially documented in the literature. A wider range of literature shows that metaphors of disease as a source domain are frequently used to portray various topics and themes related to: political unrest (Charteris-Black, 2011; Musolff, 2016; Wehling, 2016), societal problems (Woodhams, 2012; Pelosi et al., 2014; Charteris-Black, 2017), sociopolitical issues (O'Brien, 2003; Zibin, 2020), health challenges (Balteiro, 2017; Cotter et al., 2021; Olza et al., 2021), financial crisis and economic dilemmas (Charteris-Black, 2004; Negro, 2016; Brugman et al., 2019), technical problems (Isaeva and Burdina, 2019; Oster, 2019), and negative emotions (Kövecses, 2000; Zibin and Hamdan, 2023).

The scholarly investigations, the methodological designs, and the theoretical and conceptual orientations and frameworks provided several contributions, outcomes, and implications in the literature revolving around disease and disease metaphors. First and foremost, the majority of the scholarly treatments primarily associate disease and disease metaphors with the notions and hypotheses of embodiment, bodily experience, experiential knowledge, and real-world experiences (Lakoff and Johnson, 1980; Kövecses, 2010; Thibodeau et al., 2017). The notion of embodiment refers to “people’s subjective, felt experiences of their bodies in action that provide part of the fundamental grounding for language and thought.” The notions of bodily experience, experiential knowledge, and real-world experiences refer to the motivation of language and thought by human experience (Kövecses, 2010, p. 325).

Considering the account of Thibodeau et al. (2017, p. 853), disease “metaphors pervade discussions of abstract concepts and complex issues” for specific purposes and functions: (1) to facilitate communication and understanding; (2) to guide thought and behavior; (3) to influence people’s inferences, reasoning, attitudes, and evaluations; (4) to invoke specific measures, actions, policies, and support; (5) to instantiate different aspects, stages, and layers of the addressed topics such as causes, symptoms, medical examinations, medical interventions, medical prescriptions and treatments, and recovery. Moreover, metaphors that stem from disease (either as source or target domains) commonly take center stage to provide negative evaluations and attitudes; such negative evaluations and attitudes rest on certain attributes and characterizations ascribed to specific aspects of disease – e.g., threat, fear, and panic. The semantic domain of disease could also provide inductions for positive evaluations and attitudes – e.g., cure, care, recovery, compassion, and collaboration.

To summarize, disease metaphors have received scholarly attention in various different languages. Several research designs, theoretical frameworks, and methodologies were used to study these metaphors. The outcomes are as follows: Disease metaphors have been examined either as a source domain or as a target domain. These metaphors are usually related to topics associated with issues, problems, challenges, threats, risks, and unrest. They are frequently used to provide negative evaluations and attitudes, and could provide inductions for positive evaluations and attitudes. Disease metaphors are primarily associated with embodiment, bodily experience, experiential knowledge, and world real experiences.

In the light of all this, several dimensions become obvious. Although there is a growing scholarship on disease metaphors and their implications in Arabic (Zibin, 2020; Abaalalaa and Ibrahim, 2022; Zibin and Hamdan, 2023), this scholarship is not systematic and scarce, as it is also still in its infancy. Sports fanaticism and its metaphors have escaped systematic identification and operationalization among the social and political dilemmas documented and reported in the literature. Additionally, sports fanaticism and its metaphors have escaped systematic analysis in languages and cultures across the globe, in general, and Arabic, in particular. Therefore, the current study presents an unprecedented analysis of disease metaphors linked with sports fanaticism as a discursively constructed social and national problem. The following section reports on the data and methods.

## 3 Data and methods

This section presents information on the data and analytical methods and procedures utilized in this study. The section is divided into three subsections: preliminary data (3.1), the corpus (3.2), searching the corpus (3.3), and metaphor identification procedures and data analysis (3.4). The following begins with the subsection on preliminary data.

### 3.1 Preliminary data

After initially observing and noticing the metaphorical patterns in the sports articles of several Saudi newspapers, three sports articles of three Saudi newspapers were collected. The aforementioned three articles served as the preliminary data source for this research. These sports articles were all mainly obtained from three of the widely respected and widely read Saudi newspapers published online: *Alwatan* newspaper; *Okaz* newspaper; *Alyaum* newspaper. Each sports article was obtained from each newspaper.

### 3.2 The corpus

The analysis and findings of the preliminary data and the previous initial stage assisted the researchers to look for larger corpora in order to investigate the use of the disease metaphors for sports intolerance/fanaticism. Large corpora could offer a wide range of tokens, frequency, and new points of discovery. Therefore, King Abdulaziz City for Science and Technology Arabic Corpus (KACSTAC) served as the primary data source for this study. KACSTAC is a strategic initiative and a promising project to build a rich Arabic Corpus and enrich the content and knowledge of Arabic. KACSTAC contains more than a billion words. It has been designed taking into consideration several dimensions: chronology/history, geography, text type, and genre. Currently, KACSTAC contains only complete written texts, disregarding spoken and truncated texts. KACSTAC offers a number of corpus analysis tools including but not limited to: general search, specialized search (e.g., by location/country, time period, topic, field, or text type/genre), frequency distribution, concordance, and collocation.

### 3.3 Searching the corpus (KACSTAC)

Using the corpus specialized search tool and constraining the search into newspapers in Saudi Arabia, we searched KACSTAC using the word التعصب *ʔattaʕaṣṣub* ‘intolerance.’ Many instances appeared (3,168 instances within 1984 texts); however, after carefully scrutinizing all of the instances, the word *ʔattaʕaṣṣub* appeared to be used in various linguistic and discourse contexts, sometimes with collocations, to indicate several meanings (i.e., what is seen as homonyms), as the following Table 1:

**Table 1:** Meanings of the word *ʔattaʕaṣṣub* found in KACSTAC.

Intolerance	bigotry	tightening a cover one’s head
Tribalism	fanaticism	support
Racism	jealousy	arrogance
extremism	prejudice	

Considering the aims of the current research, the authors narrowed down the search in the corpus using the collocation الرياضي التعصب *ʔattaʕaṣṣub ʔarriyaẓi* ‘sports intolerance; sports fanaticism’ with the following constraints: the text type of newspapers and location in Saudi Arabia. A total number of 126 articles appeared. All articles from the corpus were extracted and downloaded in order to conduct in-depth analytical treatments and procedures of relevance to metaphor analysis.

### 3.4 Analytical methods and procedures

The gradual theoretical evolution and the continuous methodological developments of scientific inquiry related to metaphor analysis provided the literature with several proposals and procedures of metaphor analysis (Pragglejaz Group, 2007; Steen et al., 2010; Nacey et al., 2019). The analysis of metaphoricity in this research benefited from attested and empirically revealing methods of metaphor identification: the Pragglejaz Group’s metaphor identification procedure (MIP: Pragglejaz Group, 2007) and the method for linguistic metaphor identification which was refined and extended by Steen and his team at Vrije University, therefore called MIPVU (Steen et al., 2010). In addition, the discourse dynamics method of metaphor analysis (i.e., the discourse dynamics approach to metaphor) and metaphor-led discourse analysis significantly informed the processes of analysis (Cameron, 2003, 2004, 2007, 2008; Cameron et al., 2009).

The aforementioned methods of metaphor identification involved the following steps. First, after data has been downloaded, extracted, and prepared for analysis, both of the two authors read intensively (or several times) every single article on its own in order “to establish a general understanding of its meaning” (Pragglejaz Group, 2007, p. 3). This step also brought more familiarity with the data and more familiarity with aspects, forces, and constraints of the text-discourse context (Cameron et al., 2009). Since the primary goal of this study is to investigate how sports fanaticism is metaphorically represented and discursively constructed or associated with the disease or illness metaphor(s), the analysis process and identification procedures are narrowed down to specifically identify metaphoric expressions (or linguistic metaphor vehicles) associated with disease and illness used in the text-discourse contexts of sports fanaticism (the key topic).

Utilizing the Pragglejaz Group’s MIP (Pragglejaz Group, 2007) and Steen et al.’s (2010) MIPVU, (1) the articles were qualitatively, manually, and closely analyzed lexical unit by lexical unit looking for metaphoric expressions (or linguistic metaphor vehicles) associated with disease and illness. (2) Metaphoric expressions (or linguistic metaphor vehicles) associated with disease and illness were regarded, coded, highlighted, and tabulated, and metaphoric expressions which were not associated with disease and illness were disregarded as these are out of the scope of the current study. (3) We determined the contextual meaning (i.e., evoked by the text-discourse context) and the basic meaning (i.e., more concrete, related to bodily action, more precise, or historically older) of the identified linguistic metaphor vehicles. (4) We marked the lexical units as linguistic metaphor vehicles when the contextual meaning is incongruent and can be contrasted and compared with the basic meaning.

The identification and coding of the linguistic metaphor vehicles led the authors to develop a number of vehicle groupings from the data. These vehicle groupings included vehicle words or phrases according to their essential semantic meaning. Vehicle groupings were also developed by carefully observing and noticing the emergent themes and the evolving patterns in the data. It is important to mention that “the linguistic metaphor vehicle [was] the basic unit of analysis” (Cameron et al., 2009, p. 75) while the vehicle groupings were used to guide the analysts to look for patterns and systematicity across the data and the metaphors. The close examination of the patterns and connections of the linguistic metaphor vehicles and the vehicle groupings enabled the analysts to put together, trace, and categorize a number of systematic metaphors. The linguistic metaphor vehicles, vehicle groupings, and systematic metaphors are all reported and displayed in the following section (4. Findings and Analysis).

An example should clarify the analytical approach and procedures adopted for this research. For instance, the lexical unit تشنج *tašannuj* “seizure” was used to describe a type of community reaction or symptom toward/of fanaticism (i.e., the contextual meaning). According to معجم المعاني Muʕjam ʔalmaʕaanii (2023) ‘the Dictionary of Meanings’, the word تشنج *tašannuj* “seizure” is used primarily in the medical field to refer to strong and uncontrolled muscular and/or neural movements; it is a symptom of disease/illness or an injury. The previously mentioned is the basic meaning of the word تشنج *tašannuj* “seizure” in Arabic. In this case, the contextual meaning is incongruent and can be contrasted and compared with the basic meaning; hence, the word تشنج *tašannuj* “seizure” was marked metaphorical and qualified as a linguistic metaphor vehicle. Along the process of analysis, identifying other linguistic metaphor vehicles, and coding, the word تشنج *tašannuj* “seizure” was assigned into the vehicle grouping: (disease/illness) *SYMPTOMS*. This systematic pattern and use of metaphorical expressions and vehicle grouping allowed the analysts to initiate and develop the following systematic metaphor: *FANATICISM/SPORTS INTOLERANCE HAS DISEASE/ILLNESS SYMPTOMS*.

Several notes about the procedures and stages of analysis should be in order here: The analytical procedures were applied equally to both the preliminary data and the data downloaded and extracted from the corpus. The analysts kept close consideration of the context and any relevant factors, forces, or constraints through the analysis. The analytical approach can best be described as hermeneutic, hence involving recursive, interpretative, and iterative processes that informed each other and included many rounds of analysis. Trustworthiness, reliability, and rigor are enhanced through specific training and ongoing cross-rate checks at all stages of the analytical procedures and methods. The analysis and findings are offered in the next section.

## 4 Analysis and findings

This section reports the results as found in the preliminary data and the corpus throughout the analytical approach and procedures utilized to investigate the discursive construction and metaphorical framing of fanaticism as disease/illness. In the following, gives an overview of the disease/illness metaphors associated with fanaticism (Table 2).

**Table 2:** Metaphors of disease/illness associated with sports fanaticism.

Key discourse topic: Fanaticism/Sports Intolerance				
Systematic metaphor	Vehicle grouping	Vehicle Example(s)	Tokens (N)	Frequency (%)
Fanaticism is disease/illness	Disease/illness	*maraẓ* “disease/illness”	42	19.8%
Fanaticism has (disease/illness) causes	Causes	*ʔssabab* “causes”	14	6.6%
Fanaticism has (disease/illness) symptoms	Symptoms	*ʔiḥtiqan* “congestion”	69	32.5%
Fanaticism is evaluated and examined (as disease/illness)	Evaluations	*muʕdii* “infectious”	45	21.2%
Fanaticism has (disease/illness) needs for medical solutions, interventions, treatments, and/or prescriptions	Treatments	*ʕilaj* “medication”	42	19.8%
		Total	212	100%

Of course, sports fanaticism was the key discourse topic, which was under research and investigation in the current study. Importantly, the table also presents the results of systematic metaphors, vehicle groupings, and a sample of the linguistic metaphor vehicles. The table also displays the number of tokens and frequency of the linguistic metaphor vehicles associated with each systematic metaphor and each vehicle grouping.

Five systematic metaphors emerged and evolved throughout the data analysis, which suggest that fanaticism can be associated with disease/illness. Each of these systematic metaphors are demonstrated below. Examples have the following data representation: The first line presents the original Arabic text; the second line presents a transliteration of the original Arabic text; the third line presents glossing of the transliteration; the fourth line presents a translation.

### 4.1 Fanaticism is disease/illness

In this systematic metaphor, the analyzed texts addressed fanaticism using general and direct disease/illness linguistic metaphor vehicles or specific names of diseases/illnesses. Forty-two linguistic metaphor vehicles were identified in the data with a percentage of 19.8%. For instance, shows that the analysts observed the use of the following expressions as linguistic metaphor vehicles of disease) (Table 3):

**Table 3:** Linguistic metaphor vehicles of disease.

Linguistic metaphor vehicle	Translation
*maraẓ*	Disease/illness
*balaaʔ*	Disease/illness
*daaʔ*	Disease/illness
*suqm*	Disease/illness
*saraṭaan*	Disease/illness
*ʔaafah*	Lesion/disease/illness
*wabaaʔ*	Epidemic

Examples (1) and (2) demonstrate the use of these linguistic metaphor vehicles, classified under the vehicle group *DISEASE/ILLNESS* and under the systematic metaphor *FANATICISM IS DISEASE/ILLNESS*.

Example (1):

التعصب الرياضي سرطان المجتمع

ʔattaʕaṣṣub ʔarriyaẓi ***saraṭan*** ʔalmujtamaʕ

fanaticism sports ***cancer*** society

Sports fanaticism is the ***cancer*** of society

Example (2)

التعصب الرياضي مرض يؤثر في جسد العلاقة الاجتماعية

ʔattaʕaṣṣub ʔarriyaẓi ***maraẓ*** yuʔathir fii jasad ʔalʕalaqah ʔljtimaʕiyah

fanaticism sports ***disease*** influencing in body relationship social

Sports fanaticism is a ***disease*** influencing the body of social relationship

### 4.2 Fanaticism has (disease/illness) causes

In reality and practice, there are different kinds of causes for different kinds of diseases/illnesses. Fanaticism was portrayed to have its own causes as a disease/illness. Fourteen linguistic metaphor vehicles were identified in the data with a percentage of 6.6%. In the data, writers usually classify or link fanaticism to disease/illness in the beginning of the texts. Then, they refer to the causes of this disease/illness (i.e., fanaticism) using the term *ʔasbab* “causes” or *ʕawamel* “factors” while the reader or text receiver has already conceptualized fanaticism as disease/illness. Sometimes, very specific factors or causes are mentioned directly as the “causes of disease/illness” referring, of course, to fanaticism. Examples (4) and (5) illustrate the use of these metaphorical expressions, categorized under the vehicle grouping *CAUSES* and under the systematic metaphor *FANATICISM HAS (DISEASE/ILLNESS) CAUSES*.

Example (3)

الإعلام هو السبب الرئيس لانتشار هذا المرض

ʔlʕlam huwa ***ʔssabab*** ʔrraʔiis lintišar haḏa ʔalmaraẓ

media is ***cause*** major spreading this disease

Media is the major ***cause*** for spreading this disease

Example (4)

وبحث أسبابه وإيجاد طرق للحد من انتشاره...

… wa baḥth ***ʔsbaabih*** wa ʔiijad ṭuruq lilḥad min ʔintišarih

… and searching ***causes.its*** and finding ways to.limit from spread.its

… and searching its ***causes*** and finding ways to limit its spread

### 4.3 Fanaticism has (disease/illness) symptoms

A disease/illness has to have symptoms. Fanaticism in Saudi sports has been portrayed to have disease/illness symptoms as well. Note that the majority of linguistic metaphor vehicles are classified under the vehicle grouping *SYMPTOMS* and under the systematic metaphor *FANATICISM HAS (DISEASE/ILLNESS) SYMPTOMS*. Shows 69 instances of linguistic metaphor vehicles with a percentage of 32.5%. For example, writers utilized the following expressions as linguistic metaphor vehicles in the data (Table 4):

**Table 4:** Linguistic metaphor vehicles of disease symptoms.

Linguistic metaphor vehicle	Translation
*ʔaʕraẓ/muʔašširaat*	Disease/illness symptoms
*ḥumma*	FEVER
*ʔaṣabat/tuṣiib*	Injured/infected/to injure/to infect
*ʔatʕabat*	Exhausted
*ʔaʕyat*	Over-fatigued/Exhausted
*naziif*	Bleeding
*tašannuj*	Seizure
*tawattur/ẓaġt*	Pressure
*qalaq*	Anxiety
*ʔifraazaat*	Secretions
*ihtiqan*	Congestion
*ʔaʕmaa*	To blind
*ʔijhaaẓ*	Miscarriage

Examples (5) and (6) illustrate the use for a sample of these linguistic metaphor vehicles, identified under the vehicle grouping *SYMPTOMS* and under the systematic metaphor *FANATICISM HAS (DISEASE/ILLNESS) SYMPTOMS*.

Example (5)

التعصب الرياضي الذي تتعدى إفرازاته الرقعة الرياضية

ʔattaʕaṣṣub ʔarriyaẓi ʔallaḏii tataʕadda ***ʔifraazaatuh*** ʔarriqʕah ʔarriyaẓiyyah

fanaticism sports whose exceed ***secretions*** stitches sports

Sports fanaticism whose ***secretions*** exceed the sports stitches

Example (6)

وقد بلغ هذا الاحتقان ذروته من خلال العديد من مقاطع اليوتيوب

wa qad balaġa haḏa ***ʔalʔiḥtiqan*** ḏurwatuh min xilal ʔalʕadid min maqatiʕ

and has reached this ***congestion*** climax.its from through many from videos

ʔalyutyub

YouTube

And this ***congestion*** has reached its climax through many YouTube videos

### 4.4 Fanaticism is evaluated and examined (as disease/illness)

A disease/illness can be evaluated, assessed, and examined. By doing evaluations and examinations, suggestions and/or judgments are expected to be provided about the situation of the disease/illness. As such, the disease’s degree of danger, severity, and harm can be determined. The data under investigation provided a patterned use of linguistic expressions linked to disease/illness evaluations and examinations with the frequency of 45 and a percentage of 21.2%. The writers used these linguistic expressions to describe, evaluate, and examine the situation of fanaticism in the Saudi sports. The following linguistic metaphor vehicles are used to evaluate and examine fanaticism Table 5:

**Table 5:** Linguistic metaphor vehicles of medical evaluations.

Linguistic metaphor vehicle	Translation
*ʔistašraa*	Spread/transmit/outbreak
*tafaššaa*	Spread/transmit/outbreak
*ʔintašara*	Spread/transmit/outbreak
*ẓarar*	Damage
*xaṭar/xuṭuurah/maxaaṭir*	Danger/dangerous
*fattaak*	Incurable/Fatal
*ʕuẓaal*	Incurable/Fatal
*muʕdii*	Infectious
*nuʕaanii/tuʕaanii*	To suffer

Examples (7) and (8) illustrate the use for a sample of these linguistic metaphor vehicles, identified under the vehicle grouping *evaluations* and under the systematic metaphor *fanaticism is evaluated and examined (as disease/illness)*.

Example (7)

كيف يتحول أمر ترفيهي ترويحي عن النفس إلى داء خطير

kayfa yataḥawwal ʔamr tarfihi tarwiiḥii ʔan ʔannafs ʔilaa daʔ xaṭeer

how turns thing entertaining entertaining of self into disease dangerous

How a self-entertaining thing turns into a dangerous disease

ومرض عضال

wa maraẓ ***ʕuẓaal***

and disease ***fatal***

and a ***fatal*** disease

Example (8)

إطلاق هذه المبادرة أتى نظرًا لتفشي داء التعصب الرياضي

ʔiṭlaq haḏihi ʔalmubadrah ʔataa naẓaran ***litafaššii*** daʔ ʔattaʕaṣṣub ʔarriyaẓi

initiating this initiative came because.of ***outbreak*** disease fanaticism sports

Initiating this initiative came seeing the ***outbreak*** of sports fanaticism disease

### 4.5 Fanaticism has (disease/illness) needs for medical solutions, interventions, treatments, and/or prescriptions

After identifying the causes and symptoms of the disease and after examining, evaluating, and judging the disease situation, the disease needs medical solutions, interventions, treatments, and/or prescriptions. The writers also associated fanaticism with medical solutions, interventions, treatments, and prescriptions; thus, depicting fanaticism as a disease. A number of 42 tokens with a percentage of 19.8% were found in the data. The data analysis offered the following linguistic metaphor vehicles Table 6:

**Table 6:** Linguistic metaphor vehicles of medical treatments.

Linguistic metaphor vehicle	Translation
*ʔaxissaaʔiyiin*	Medical specialists
*muʕalajah/ʕilaj*	Medication/to medicate/to prescribe medicine
*mukaafaha*	Medication/to medicate/to prescribe medicine
*batr*	Amputation/to amputate
*riqʕah*	Stitch
*weqaayah*	Protection

Examples (9) and (10) illustrate the use for a sample of these linguistic metaphor vehicles, identified under the vehicle grouping *treatments* and under the systematic metaphor *fanaticism has (disease/illness) needs for medical solutions, interventions, treatments, and/or prescriptions*.

Example (9)

علاج التعصب الرياضي لدى مركز الحوار الوطني

***ʕilaj*** ʔattaʕaṣṣub ʔarriyaẓi ladaa markaz ʔalḥiwar ʔalwatanii

***medicine*** fanaticism sports has Center Dialogue National

The National Dialogue Center has the ***medicine*** of sports fanaticism

Example (10)

واستمرار الحلم لا يعني عدم القدرة على بتر الداء

wa ʔistimrar ʔlḥulum laa yaʕnii ʕadam ʔalqudrah ʕalaa ***batr*** ʔaddaʔ

and pursuing dream not mean not ability on ***amputate*** disease

And pursuing the dream does not mean the inability to ***amputate*** the disease

To summarize, the analysis and findings empirically prove the existence, usage, and productivity of disease metaphors in the discursive construction and metaphorical framing of sports fanaticism in the Saudi newspapers discourse. This current section demonstrates, with samples and examples, the linguistic metaphor vehicles, vehicle groupings, and systematic disease metaphors linked with the key discourse topic of sports fanaticism. The following section provides a discussion of the analysis and findings.

## 5 Discussion

This section provides explanations, interpretations, and discussions for the construction, appearance, and use of the disease metaphors to portray sports fanaticism in the Saudi newspapers discourse. Before delving more, it is very important to mention the following about construing fanaticism as disease: This metaphor can be seen as emergent; it can also be described in terms of prominence and sophistication; it is patterned and systematic. The adoption of the discourse dynamics approach to metaphor and metaphor-led discourse analysis seems to offer and afford an analytical power, which significantly helped to unpack the emergence, prominence, sophistication, and systematicity of relevance to the use of this metaphor. Additionally, this theoretical framework with its analytical toolkit enabled the analysts to build good views about the ideas, attitudes, and values connected with sports fanaticism and disease metaphors.

The findings shown and demonstrated in the previous section might seem to align and can be related with other results and discussions reported in the literature about the use and perpetuation of the disease metaphors across and within different kinds of discourse (Balteiro, 2017; Charteris-Black, 2017; Potts and Semino, 2019; Cotter et al., 2021, *inter alia*). The findings are fairly close with the systematic metaphors: *fanaticism is disease/illness, fanaticism is evaluated and examined (as disease/illness), fanaticism has (disease/illness) needs for medical solutions, interventions, treatments, and/or prescriptions.* The systematic metaphor *fanaticism has (disease/illness) causes* appeared the least in frequency. Perhaps, this can be seen as real and literal causes are the least visible aspect of disease. However, the systematic metaphor *fanaticism has (disease/illness) symptoms* is noticeably the most frequently used one. Of course, disease symptoms, evaluations, and treatments are more visible, tangible, and concrete. Overall, these findings may signal an ongoing social and national dilemma of struggle and suffer from sports fanaticism in the Saudi nation.

Metaphors are used to simplify complex, abstract, and technical concepts and knowledge and to structure our understanding of the world (Charteris-Black, 2004; Thibodeau and Boroditsky, 2011; Burgers et al., 2016; Balteiro, 2017; Thibodeau et al., 2017; Hendricks et al., 2018). It seems that the concept of fanaticism is a complex and abstract one. In this regard, the disease among other metaphors can have the capacity to facilitate conceptualizing and thinking about complex concepts and abstract issues. Conceptualizing fanaticism in terms of disease establishes certain perspectives and enforces new ways of understanding which, in turn, dynamically influences our perception of fanaticism. These perspectives and understandings develop a certain sense of reality and indicate a dynamic process of meaning-making leading to the production and, subsequently, the provision and manifestation of the metaphorical frames and systematic metaphors in the analyzed discourse and the reported findings.

The results and analysis can also be related to “salient aspects of people’s experiences of real world” disease and other health issues (Johansson Falck, 2018, p.63). That is, the use of linguistic metaphor vehicles of disease in this study suggests a reflection of a universal bodily experience commensurate with shared knowledge and common ground, which can be shared by and traced in different languages (Deignan and Potter, 2004; Balteiro, 2017; also see Kövecses, 2000, 2005, 2010) such as English (Hendricks et al., 2018). The reported systematic metaphors, with groupings and linguistic metaphor vehicles, offer an interesting reconstruction for the significant and main aspects of a real world experience of disease. As shown, such an experience for a sick person prototypically has the following stages: causes of disease, symptoms of disease, medical evaluations, and medical treatments. The disease metaphors and their variants seem conventionalized and universal of human embodied experiences and human shared cultural knowledge. This previously mentioned real world and bodily experience of disease can be considered a factor which has led for the establishment and the emergence of the disease systematic metaphors and figurative frames in the Saudi newspapers discourse addressing fanaticism.

Although, as Potts and Semino (2019) mentioned, many metaphors seem conventionalized, “metaphor choices are seldom neutral” (Potts and Semino, 2019, p. 81). Metaphors can be used to frame different kinds of phenomena in order to facilitate different understandings and evaluations. The metaphoricity and framings associated with disease are no exception. On one hand, these metaphors are mostly and primarily associated with negative connotations and evaluations. They are used in the topics of social disorders and problems. As shown in the findings, all the identified linguistic metaphor vehicles carry disease and health connotations. When fanaticism is framed in terms of disease, a number of reflections, evaluations, and appraisals start to appear and become salient. These metaphorical frames seem to encourage the audience to appraise and evaluate the seriousness and severity of the situation. Therefore, if something or a situation such as fanaticism is described in terms of disease metaphorical frames, such a tendency, using Sontag’s (1979) words, might suggest that the situation is “unqualifiedly and unredeemably wicked” (p. 83) and develops an “incitement to violence” since it “encourages fatalism and justifies severe measures” (p. 84). On the other hand, disease metaphorical frames can invite positive ideas, attitudes, and values which positively influence people’s inferences, reasoning, and evaluations. Hence, disease metaphors can evoke such positive aspects of cure, care, recovery, compassion, collaboration, sympathy, and pity. The functional aspects of the discourse might influence the negative or positive evaluations of disease metaphorical frames (Steen et al., 2010; Cotter et al., 2021).

The current analysis majorly concentrated on newspapers discourse and news texts. This type of discourse and texts are characterized by deliberateness, selectivity, and general world knowledge (Biber, 1988; Richardson, 2007; Steen et al., 2010; Sowińska, 2013). According to Steen et al. (2010), pp. 43–44; see also Biber, 1988; Richardson, 2007; Sowińska, 2013), “[t]he news production process allows journalists to carefully craft their texts and make precise lexical choices.” As such, it seems that the Saudi newspapers discourse has deliberately and strategically constructed, co-constructed, and reconstructed the disease metaphors to refer to fanaticism. That being said brings about the following question: Why have Saudi newspapers or writers used the metaphors of disease in order to project fanaticism in this specific time, in this specific place (i.e., Saudi Arabia), and through this specific medium (i.e., newspapers)? One view, we suggest, is that the writers wanted to view fanaticism as gradable (Sowińska, 2013); to illustrate, the newspapers choice of the disease metaphors is used to portray the severity as well as to give an easily and tangibly conceptualized message about the intense negative degree (Todolí, 2007; García, 2010; Trckova, 2012) of fanaticism being a social issue in Saudi Arabia. Moreover, the image of disease has been employed in the Saudi newspapers discourse to bring attention to the terrible consequences (Todolí, 2007; García, 2010) that fanaticism have had and still to be expected to have in the future, to name a few according to some sports news reports: ending friendships; causing arguments which develop into fights between friends, even family members, using different kinds of weapons; causing divorce cases. Consequently, such a metaphor, using Trckova’s (2012), p.146) words, “has the main effect of demonizing” fanaticism in Saudi Arabia.

It is also important to not overlook the role of the audience in consuming and digesting, thereafter, influenced by newspapers discourse (Richardson, 2007). That is, newspapers discourse is recipient-designed and audience-oriented. According to Richardson (2007, p. 29), “journalistic discourse, in particular, is one active element in bringing about [a] change through shaping understandings, influencing audience attitudes and beliefs (particularly through their reinforcement), and transforming the consciousness of those who read and consume it.” Richardson (2007, p. 7) added, “journalism exists to enable citizens to better understand their lives and their position(s) in the world.” In line with these assumptions, it seems that the newspapers ascribed disease to fanaticism in order to appeal to the public (Ferrari, 2007; Todolí, 2007; Sahlane, 2013), so people can help limit and control this social issue of sports intolerance, leading to reduce its consequences. In similar perspectives to that of Sahlane (2013) and Sowińska (2013), associating fanaticism with disease metaphors encourages the audience to be patient since there are no immediate and magical expected results out of disease treatments; rather, diseases are mostly treated on a long-term basis which in turn encourages the audience to accept suffering and struggle. Some diseases can never completely be cured such as kinds of cancer. Therefore, some diseases are life-threatening.

## 6 Conclusion

Newspaper articles obtained from the corpus appeared as a productive source of texts and figurative language that provided data related to the topic of sports fanaticism in Saudi Arabia. The search hits in the corpus proved that to be so. Sports fanaticism seems to be a fast-developing social problem in Saudi Arabia. It also seems that fanaticism is a hardship and a national dilemma considering the different reports about such a phenomenon. It is important to mention that the topic, data, and type of data under investigation in the current research is part and parcel of larger discourses on the national level of Saudi Arabia, all of which are targeted to provide awareness and other specific measures in order to decrease any consequences of this social issue and national problem. The findings and analysis demonstrated several points: the emergence of sports fanaticism as a topic and disease metaphors is shaped and developed by the societal context and the nature of newspapers texts and discourse; it is enriched and influenced by the discourse situation and the needs to jointly construct and communicate intense experiences through specific evaluations and referential functions, which have a powerful resonance for the Saudi national and social levels; the use of disease metaphorical frames to structure our understanding and to simplify the concept of fanaticism. For future research, analysis and demonstration should include other metaphorical frames linked with the topic of sports fanaticism. New sources and forms of data would provide novel contributions and enrichment.

## Data availability statement

The original contributions presented in the study are included in the article/supplementary material, further inquiries can be directed to the corresponding author.

## Author contributions

YM: Conceptualization, Data curation, Formal analysis, Funding acquisition, Investigation, Methodology, Writing – original draft, Writing – review & editing. FA: Data curation, Investigation, Methodology, Project administration, Resources, Validation, Writing – review & editing.

## References

[ref1] Abaalalaa H. Ibrahim W. (2022). Metaphors of cancer in the Arabic language: an analysis of the use of metaphors in the online narratives of breast cancer patients. Open Linguist. 8, 27–45. doi: 10.1515/opli-2022-0184

[ref2] Bakhtiar M. (2017). The role of context in the formation ofhejab ‘veiling’ metaphors inhejabbillboards and posters in Iran. Metaphor Soc. World 7, 159–189. doi: 10.1075/msw.7.2.01bak

[ref3] Balteiro I. (2017). Metaphor in Ebola’s popularized scientific discourse. Iberica 34, 209–230.

[ref4] Bates B. (2020). The (in) appropriateness of the WAR metaphor in response to SARS-CoV-2: a rapid analysis of Donald J. Trump's rhetoric. Front. Commun. 5, 1–12. doi: 10.3389/fcomm.2020.00050

[ref5] Biber D. (1988) Variation across speech and writing. Cambridge: Cambridge University Press.

[ref6] Bowker J. (1996). Cancer, individual process, and control: a case study in metaphor analysis. Health Commun. 8, 91–104. doi: 10.1207/s15327027hc0801_5

[ref7] Brugman B. C. Burgers C. Vis B. (2019). Metaphorical framing in political discourse through words vs. concepts: a meta-analysis. Lang. Cogn. 11, 41–65. doi: 10.1017/langcog.2019.5

[ref8] Brugman B. Droog E. Reijnierse W. Leymann S. Frezza G. Renardel de Lavalette K. (2022). Audience perceptions of COVID-19 metaphors: the role of source domain and country context. Metaphor. Symb. 37, 101–113. doi: 10.1080/10926488.2021.1948332

[ref003] Burgers C. Konijn E. Steen G. (2016). Figurative framing: Shaping public discourse through metaphor, hyperbole, and irony. Commun Theory. 26, 410–430. doi: 10.1111/comt.12096

[ref9] Cameron L. (2003). Metaphor in educational discourse. London; New York, NY: Continuum.

[ref10] Cameron L. (2004). Metaphor clusters in discourse. J. Appl. Linguist. 1, 107–136. doi: 10.1558/japl.2004.1.2.107

[ref11] Cameron L. (2007). Patterns of metaphor use in reconciliation talk. Discourse Soc. 18, 179–222. doi: 10.1177/0957926507073376

[ref12] Cameron L. (2008). “Metaphor in the construction of a learning environment” in Metaphors for learning. ed. Berendt E. (Amsterdam: John Benjamins)

[ref13] Cameron L. Maslen R. Todd Z. Maule J. Stratton P. Stanley N. (2009). The discourse dynamics approach to metaphor and metaphor-led discourse analysis. Metaphor. Symb. 24, 63–89. doi: 10.1080/10926480902830821

[ref14] Charteris-Black J. (2004). Corpus approaches to critical metaphor analysis. Basingstoke, UK: Palgrave Macmillan. doi: 10.1057/9780230000612

[ref15] Charteris-Black J. (2011). Politicians and rhetoric: The persuasive power of metaphor. New York: Palgrave Macmillan.

[ref16] Charteris-Black J. (2017). Fire metaphors: Discourses of awe and authority. London: Bloomsbury.

[ref17] Chiang W. Duann R. (2007). Conceptual metaphors for SARS: “war” between whom? Discourse Soc. 18, 579–602. doi: 10.1177/0957926507079631

[ref18] Cotter C. Samos D. Swinglehurst D. (2021). Framing obesity in public discourse: representation through metaphor across text type. J. Pragmat. 174, 14–27. doi: 10.1016/j.pragma.2020.12.015

[ref19] Craig D. (2020). Pandemic and its metaphors: Sontag revisited in the COVID-19 era. Eur. J. Cult. Stud. 23, 1025–1032. doi: 10.1177/1367549420938403

[ref20] Deignan A. Potter L. (2004). A corpus study of metaphors and metonyms in English and Italian. J. Pragmat. 36, 1231–1252. doi: 10.1016/j.pragma.2003.10.010

[ref21] Dilai M. Serafin T. (2019). “Metaphorical conceptualization in the Euromaidan discourse” in Current approaches to metaphor analysis in discourse. ed. Ferrando I. (Berlin/Boston: De Gruyter)

[ref22] Ferrari F. (2007). Metaphor at work in the analysis of political discourse: investigating a ‘preventive war' persuasion strategy. Discourse Soc. 18, 603–625. doi: 10.1177/0957926507079737

[ref23] Flusberg S. Matlock T. Thibodeau P. (2018). War metaphors in public discourse. Metap. Symb. 33, 1–18. doi: 10.1080/10926488.2018

[ref004] García M. (2010). Diagnosing terrorism in Spain: Medical metaphors in presidential discourse. Southwest J. Linguist. 29, 53–73.

[ref24] Goatly A. (2007). Washing the brain: Metaphor and hidden ideology. Amsterdam/Philadelphia: John Benjamins Publishing.

[ref25] Gwyn R. (1999). ““Captain of my own ship”: metaphor and the discourse of chronic illness” in Researching and applying metaphor. eds. Cameron L. Low G. (Cambridge: Cambridge University Press)

[ref26] Hanne M. Hawken S. J. (2007). Metaphors for illness in contemporary media. Med. Humanit. 33, 93–99. doi: 10.1136/jmh.2006.000253 23674429

[ref27] Hauser D. J. Schwarz N. (2015). The war on prevention: bellicose cancer metaphors hurt (some) prevention intentions. Personal. Soc. Psychol. Bull. 41, 66–77. doi: 10.1177/0146167214557006 25352114

[ref28] Hauser D. J. Schwarz N. (2019). The war on prevention II: Battle metaphors undermine cancer treatment and prevention and do not increase vigilance. Health Commun. 35, 1698–1704. doi: 10.1080/10410236.2019.1663465 31496298

[ref29] Hendricks R. K. Demjén Z. Semino E. Boroditsky L. (2018). Emotional implications of metaphor: consequences of metaphor framing for mindset about cancer. Metaphor. Symb. 33, 267–279. doi: 10.1080/10926488.2018.1549835

[ref30] Hudson C. (2008). “Singapore at war: SARS and its metaphors” in The social construction of SARS: Studies of a health communication crisis. eds. Powers J. H. Xiao X. S. (Amsterdam: John Benjamins Publishing)

[ref31] Isaeva E. Burdina O. (2019). “Transdiscursive term transformation: the evidence from cognitive discursive research of the term ‘virus’” in Current approaches to metaphor analysis in discourse. ed. Ferrando I. (Berlin/Boston: De Gruyter)

[ref32] Johansson Falck M. (2018). From ecological cognition to language: when and why do speakers use words metaphorically? Metaphor. Symb. 33, 61–84. doi: 10.1080/10926488.2018.1434937

[ref33] Koteyko N. Brown B. Crawford P. (2008). The dead parrot and the dying swan: the role of metaphor scenarios in UK press coverage of avian flu in the UK in 2005–2006. Metaphor. Symb. 23, 242–261. doi: 10.1080/10926480802426787

[ref34] Kövecses Z. (2000). Metaphor and emotion: Language, culture, and body in human feeling. Cambridge: Cambridge University Press.

[ref35] Kövecses Z. (2005). Metaphor in culture: Universality and variation. Cambridge: Cambridge University Press.

[ref36] Kövecses Z. (2010). Metaphor: A practical introduction. Oxford, UK: Oxford University Press.

[ref37] Lakoff G. Johnson M. (1980). Metaphors we live by. Chicago: University of Chicago Press.

[ref38] Landtsheer C. (2009). “Collecting political meaning from the count of metaphor” in Metaphor and discourse. eds. Musolff A. Zinken J. (Basingstoke/New York: Palgrave Macmillan)

[ref39] Larson B. Nerlich B. Wallis P. (2005). Metaphors and biorisks: the war on infectious diseases and invasive species. Sci. Commun. 26, 243–268. doi: 10.1177/1075547004273019

[ref40] Lu H. Schuldt J. (2018). Communicating Zika risk: using metaphor to increase perceived risk susceptibility. Risk Anal. 38, 2525–2534. doi: 10.1111/risa.12982 29486059

[ref41] MacArthur F. Oncins-Martínez J. (2012). “Introduction: metaphor in use” in Metaphor in use: Context, culture, and communication. eds. MacArthur F. Oncins-Martínez J. Sánchez-García M. Piquer-Píriz A. (Amsterdam/Philadelphia: John Benjamins Publishing)

[ref42] Magaña D. Matlock T. (2018). How Spanish speakers use metaphor to describe their experiences with cancer. Discourse Commun. 2, 627–644. doi: 10.1177/1750481318771446

[ref001] Mongoven A. (2006). The war on disease and the war on terror: A dangerous metaphorical nexus?. Camb. Q. Healthc. Ethics. 15, 403–416. 17066766 10.1017/s0963180106060518

[ref43] Musolff A. (2016). Political metaphor analysis: Discourse and scenarios. London/New York: Bloomsbury.

[ref44] Muʕjam ʔalmaʕaanii (2023). معجم المعاني (lit. the Dictionary of Meanings). Available at: https://www.almaany.com/.

[ref002] Nacey S. Dorst S. Krennmayr T. Reijnierse W. (2019). Metaphor identification in multiple languages: MIPVU around the world. Amsterdam/Philadelphia: John Benjamins Publishing.

[ref45] Negro I. (2016). The human being as the target of crisis metaphors in English, Spanish and French. Metaph. Soc. World 6, 177–204. doi: 10.1075/msw.6.2.01neg

[ref46] Nerlich B. (2004). War on foot and mouth disease in the UK, 2001: towards a cultural understanding of agriculture. Agric. Hum. Values 21, 15–25. doi: 10.1023/B:AHUM.0000014022.42425.a9

[ref47] Nerlich B. Halliday C. (2007). Avian flu: the creation of expectations in the interplay between science and the media. Sociol. Health Illn. 29, 46–65. doi: 10.1111/j.1467-9566.2007.00517.x 17286705

[ref48] Nerlich B. Koteyko N. (2009). “MRSA - portrait of a superbug: a media drama in three acts” in Metaphor and discourse. eds. Musolff A. Zinken J. (Basingstoke/New York: Palgrave Macmillan), 153–169.

[ref49] O'Brien G. (2003). Indigestible food, conquering hordes, and waste materials: metaphors of immigrants and the early immigration restriction debate in the United States. Metaphor. Symb. 18, 33–47. doi: 10.1207/S15327868MS1801_3

[ref50] Olza I. Koller V. Ibarretxe-Antuñano I. Pérez-Sobrino P. Semino E. (2021). The# ReframeCovid initiative: from twitter to society via metaphor. Metaph. Soc. World 11, 98–120. doi: 10.1075/msw.00013.olz

[ref51] Oster U. (2019). “Cross-cultural semantic and pragmatic profiling of emotion words. Regulation and expression of anger in Spanish and German” in Current approaches to metaphor analysis in discourse. ed. Ferrando I. (Berlin/Boston: De Gruyter)

[ref52] Pelosi A. de Moraes Feltes H. P. Cameron L. Cameron L. (2014). Urban violence in Brazil and the role of the media: communicative effects of systematic metaphors in discourse. Metap. Soc. World 4, 27–47. doi: 10.1075/msw.4.1.02pel

[ref53] Pérez-Sobrino P. Semino E. Ibarretxe-Antuñano I. Koller V. Olza I. (2022). Acting like a hedgehog in times of pandemic: metaphorical creativity in the #ReframeCovid collection. Metaphor. Symb. 37, 127–139. doi: 10.1080/10926488.2021.1949599

[ref54] Periyakoil V. S. (2008). Using metaphors in medicine. J. Palliat. Med. 11, 842–844. doi: 10.1089/jpm.2008.9885 18715175

[ref55] Perrez J. Reuchamps M. (2014). Deliberate metaphors in political discourse: the case of citizen discourse. Metaphorik.de 25, 7–41.

[ref56] Pinelli E. (2016). The role of metaphor and metonymy in framing terrorism: the case of the Beslan school siege in the Russian media. Metap. Soc. World 6, 134–155. doi: 10.1075/msw.6.1.06pin

[ref57] Potts A. Semino E. (2019). Cancer as a metaphor. Metaphor. Symb. 34, 81–95. doi: 10.1080/10926488.2019.1611723

[ref58] Pragglejaz Group (2007). MIP: a method for identifying metaphorically used words in discourse. Metap. Symb. 22, 1–39. doi: 10.1080/10926480709336752

[ref59] Ribeiro B. Hartley S. Nerlich B. Jaspal R. (2018). Media coverage of the Zika crisis in Brazil: the construction of a ‘war’ frame that masked social and gender inequalities. Soc. Sci. Med. 200, 137–144. doi: 10.1016/j.socscimed.2018.01.023 29421460

[ref60] Richardson J. (2007). Analyzing newspapers: An approach from critical discourse analysis. London: Springer.

[ref61] Sabucedo J. Alzate M. Hur D. (2020). COVID-19 and the metaphor of war (COVID-19 y la metáfora de la guerra). Int. J. Soc. Psychol. 35, 618–624. doi: 10.1080/02134748.2020.1783840

[ref62] Sahlane A. (2013). Metaphor as rhetoric: newspaper Op/Ed debate of the prelude to the 2003 Iraq war. Crit. Discourse Stud. 10, 154–171. doi: 10.1080/17405904.2012.736397

[ref63] Scherer A. M. Scherer L. D. Fagerlin A. (2015). Getting ahead of illness: using metaphors to influence medical decision making. Med. Decis. Mak. 35, 37–45. doi: 10.1177/0272989X14522547 24615273

[ref64] Semino E. Demjén Z. (Eds.). (2017). The Routledge handbook of metaphor and language. Abingdon: Routledge.

[ref65] Semino E. Demjén Z. Demmen J. (2018). An integrated approach to metaphor and framing in cognition, discourse, and practice, with an application to metaphors for cancer. Appl. Linguis. 39, 625–645.

[ref66] Semino E. Demjén Z. Demmen J. Koller V. Payne S. Hardie A.. (2017). The online use of violence and journey metaphors by patients with cancer, as compared with health professionals: a mixed methods study. BMJ Support. Palliat. Care 7, 60–66. doi: 10.1136/bmjspcare-2014-000785, PMID: 25743439 PMC5339544

[ref67] Semino E. Heywood J. Short M. (2004). Methodological problems in the analysis of metaphors in a corpus of conversations about cancer. J. Pragmat. 36, 1271–1294. doi: 10.1016/j.pragma.2003.10.013

[ref68] Sontag S. (1979). Illness as metaphor. New York: Farrar, Straus, Giroux.

[ref69] Sontag S. (1989). AIDS and its metaphors. New York, NY: Farrar, Straus, and Giroux.

[ref70] Sowińska A. (2013). A critical discourse approach to the analysis of values in political discourse: the example of freedom in president Bush’s state of the union addresses (2001–2008). Discourse Soc. 24, 792–809. doi: 10.1177/0957926513486214

[ref71] Steen G. Dorst A. Herrmann J. Kaal A. Krennmayr T. Pasma T. (2010). A method for linguistic metaphor identification: From MIP to MIPVU (14). Amsterdam/Philadelphia: John Benjamins Publishing.

[ref72] Stern J. (2000). Metaphor in context. Cambridge: The MIT Press.

[ref73] Thibodeau P. H. Boroditsky L. (2011). Metaphors we think with: the role of metaphor in reasoning. PLoS One 6:e16782. doi: 10.1371/journal.pone.0016782 21373643 PMC3044156

[ref74] Thibodeau P. Hendricks R. Boroditsky L. (2017). How linguistic metaphor scaffolds reasoning. Trends Cogn. Sci. 21, 852–863. doi: 10.1016/j.tics.2017.07.001 28789831

[ref75] Todolí J. (2007). Disease metaphors in urban planning. Critic. Approach. Disc. Anal. Disc. 1, 51–60.

[ref76] Trckova D. (2012). Metaphorical representation of a natural phenomenon in newspaper discourse on natural catastrophes. Critic. Approach. Disc. Anal. Disc. 5, 137–151.

[ref77] Tsakona V. (2012). The Greek state and the plaster cast: from the Greek military junta of 21 April 1967 to the IMF and EU’s rescue mechanism. Metaph. Soc. World 2, 61–86. doi: 10.1075/msw.2.1.04tsa

[ref78] Wackers D. Y. Plug H. J. Steen G. J. (2021). “For crying out loud, don't call me a warrior”: standpoints of resistance against violence metaphors for cancer. J. Pragmat. 174, 68–77. doi: 10.1016/j.pragma.2020.12.021

[ref79] Wallis P. Nerlich B. (2005). Disease metaphors in new epidemics: the UK media framing of the 2003 SARS epidemic. Soc. Sci. Med. 60, 2629–2639. doi: 10.1016/j.socscimed.2004.11.031, PMID: 15814187 PMC7117051

[ref80] Washer P. (2004). Representations of SARS in the British newspapers. Soc. Sci. Med. 59, 2561–2571. doi: 10.1016/j.socscimed.2004.03.038, PMID: 15474209 PMC7127057

[ref81] Wehling E. (2016). “Moral disgust at its best: the important role of low-level mappings and structural parallelism in political disgust and disease metaphors” in Metaphor and communication. eds. Gola E. Evras F. (Amsterdam/Philadelphia: John Benjamins Publishing)

[ref82] Williams Camus J. (2009). Metaphors of cancer in scientific popularization articles in the British press. Discourse Stud. 11, 465–495. doi: 10.1177/1461445609105220

[ref83] Woodhams J. M. (2012). A journey towards employment. Metaph. Soc. World 2, 41–60. doi: 10.1075/msw.2.1.03woo

[ref84] Zibin A. (2020). A corpus-based study of metaphors used to describe Syrian refugees in Jordanian politico-economic discourse. Pragmat. Soc. 11, 640–663. doi: 10.1075/ps.17037.zib

[ref85] Zibin A. Hamdan J. (2023). The conceptualisation of FEAR through conceptual metonymy and metaphor in Jordanian Arabic. Int. J. Arabic Eng. Stud. 19, 239–262. doi: 10.33806/ijaes2000.19.2.1

[ref86] Zinken J. Musolff A. (2009). “A discourse-centered perspective on metaphorical meaning and understanding” in Metaphor and discourse. eds. Musolff A. Zinken J. (New York: Palgrave Macmillan)

